# Global Positioning System Analysis of Physical Demands in Elite Women’s Beach Handball Players in an Official Spanish Championship

**DOI:** 10.3390/s21030850

**Published:** 2021-01-27

**Authors:** Juan Antonio Sánchez-Sáez, Javier Sánchez-Sánchez, Alejandro Martínez-Rodríguez, José Luis Felipe, Jorge García-Unanue, Daniel Lara-Cobos

**Affiliations:** 1 GDOT Research Group, Faculty of Sport, Universidad Católica de Murcia, 30107 Murcia, Spain; jasanchez419@ucam.edu; 2 School of Sport Sciences, Universidad Europea de Madrid, 28670 Madrid, Spain; joseluis.felipe@universidadeuropea.es; 3 Department of Analytical Chemistry, Nutrition and Bromatology, Faculty of Science, Universidad de Alicante, 03690 Alicante, Spain; amartinezrodriguez@gcloud.ua.es; 4 IGOID Research Group, Physical Activity and Sport Sciences Department, Universidad de Castilla-La Mancha, 45071 Toledo, Spain; jorge.garciaunanue@uclm.es; 5 Italian Handball Federation, Stadio Olimpico (Curva Nord), 00135 Roma, Italy; daniel.lara.cobos@gmail.com

**Keywords:** tracking system, match monitoring, internal and external load, female sports teams, physical performance

## Abstract

This cross-sectional study aims to analyze the physical demands of elite beach handball players during an official competition. Nine elite female (mean age: 24.6 ± 4.0 years; body weight: 62.4 ± 4.6 kg; body height: 1.68 ± 0.059 m; training experience: 5 years; training: 6 h/week) beach handball players of the Spanish National Team were recruited for this study. A Global Positioning System was incorporated on each player’s back to analyze their movement patterns. Speed and distance were recorded at a sampling frequency of 15 Hz, whereas acceleration was recorded at 100 Hz by means of a built-in triaxial accelerometer. The main finding of the study is that 53% of the distance travelled is done at speeds between 1.5 and 5 km/h and 30% of the distance is between 9 and 13 km/h (83% of the total distance covered), which shows the intermittent efforts that beach handball involves at high intensity, as reflected in the analysis of the internal load with 62.82 ± 14.73% of the game time above 80% of the maximum heart rate. These data help to orientate training objectives to the physical demands required by the competition in order to optimize the players’ performance.

## 1. Introduction

Beach handball is a discipline that was born on the beaches of Italy in 1992 with the intention of promoting court handball during the summer period [1,2]. From the beginning, scientific studies have analyzed psychological profiles, levels of competitive anxiety or moods and self-efficacy [3], decision-making at a tactical level [4,5], body composition and anthropometric profile [6], or physical and physiological demands of beach handball players [7,8,9]. This sporting discipline is characterized by intermittent efforts, with actions lasting 6–15 s, although these physical demands vary depending on the sex of the players [5,10]. High-intensity actions predominate in this sport, with short recovery periods and constant changes in the role of the players during the game [11]. The match is divided into two sets of 10 min of duration, with the team that achieves victory in both sets being the winner. If there is a tie, a tiebreaker set is played to determine the winning team; therefore, it is necessary to know the physical demands of the player during each of the sets of the match [12,13].

Thus, the study of physical demands during sports practice has become a fundamental aspect in the design of training sessions and the assessment of physical load during competition [8,9,14]. For this, some authors differentiate between the internal load of the athlete and the external load [15]. The monitoring of this physical load has evolved in recent years based on advances in tracking technologies, such as global positioning systems (GPS) [16]. This technology makes it possible to monitor the individual physical demands of the players, as well as the collective tactical behavior within a team during training and competition [17,18]. GPS technology is an effective tool for evaluating the external load of athletes (e.g., jumps, impacts, or collisions), especially for team sports disciplines (e.g., beach football, futsal, rugby, hurling, or cricket) in the open air that incorporate a high complexity of movements. These tracking systems make it possible to identify the specific physical and physiological demands of a sport, positional determinants and collective interaction within the team, the mechanical load accumulated by the athlete, and the type of efforts that characterize a sport [17,18]. Specifically, these demands have also been analyzed in female categories (e.g., camogie, football, beach handball, or hockey) [8,9,19,20,21,22,23]. Finally, these devices can be synchronized with heart rate monitoring systems in order to relate the external load to which the athlete is subjected with the internal response derived from exercise [24,25]. In the same vein, the Abalakov jumps, Countermovement Jumps (CMJs), and Handgrip (muscular strength) tests also offer the possibility of analyzing the onset of fatigue [26].

GPS is a satellite radio navigation system with technology that allows for the monitoring of the three-dimensional movement of a subject or group in different outdoor environments [16,17,18]. This technology makes it possible to determine in real time, by triangulation [16], the positioning (spatial coordinates), 24 h a day, anywhere on earth, and under any atmospheric condition of both static and moving points with an error rate of a few meters [17,27,28]. In reference to the validity and reliability of the use of GPS technology, different studies determine an acceptable degree to evaluate the patterns of movement at lower speeds [16,18,29]. In contrast, reliability is questioned when analyzing short-distance, high-speed straight-line runs and efforts involving changes of direction (GPS with a sampling frequency of 1–5 Hz). However, GPS units with a higher sampling frequency, such as the one used in this research (15 Hz), obtain greater reliability and validity in their application in monitoring team sports, being able to measure the smallest change in the variables analyzed [16,18,30]. In this respect, it is important to consider the atmospheric conditions and the number of satellites connected simultaneously during the measurement, as they could affect the accuracy of the signal reception [17]. The evolution of this tracking technology has allowed for the inclusion of other measurement tools within these devices, such as gyroscopes and accelerometers that allow the analysis of acceleration and deceleration variables, jumps, turns, impacts (bumps or falls while standing), or changes of direction. This information provides a comprehensive analysis of the external load of athletes through the interaction of all variables, obtaining parameters such as player load or total body load that have been validated in previous studies [17,31]. The monitoring of athletes during training helps us know to what degree the training sessions replicate the physical and physiological demands of the players during competition [17,32]. However, on some occasions, the specific regulations of the sporting modality prevent the use of the GPS system in official competition, so this information can only be obtained in non-official competitions [17].

The use of this technology in beach handball for monitoring physical demands during competition is limited. Previous studies only analyzed the distance travelled and the analysis of the internal load of the player using heart rate monitoring devices [8,9]. These studies show total distances of 1118.2 ± 221.8 m and maximum speeds of 18.5 km·h^−1^ during competition in elite players, with moderate–high intensity movements. On the other hand, Zapardiel and Asín-Izquierdo [9] revealed significant differences depending on the playing position in the physical demands required during the competition. However, the players kept the same playing position during the 10 min of the set in this study. The research did not analyze official competitions, so there is a lack of information on the physical demands of beach handball players during competition. Therefore, the objective of this study is to analyze the physical demands of beach handball during competition by applying GPS technology in elite female players. The results of this study will help to orientate the training objectives to the physical demands of the competition with the aim of optimizing the performance of the players.

## 2. Materials and Methods

### 2.1. Experimental Approach to the Problem

A cross-sectional study was carried out analyzing physical and physiological parameters of elite women’s beach handball players. This study was conducted during the Spanish Beach Handball Tour (official competition).

### 2.2. Subjects

Nine elite female (mean age: 24.6 ± 4.0 years; body weight: 62.4 ± 4.6 kg; body height: 1.68 ± 0.059 m; training experience: 5 years, training: 6 h/week) beach handball players of the Spanish National Team were recruited for this study. All the beach handball players were previously informed about the objectives of the study, methods, and risks of the research and they provided informed written consent to be part of the research. The Ethics Committee at the European University of Madrid (Spain) approved the methodology of the study according to the Declaration of Helsinki.

### 2.3. Procedures

The study was conducted during the Spanish Beach Handball Tour held in Orihuela-Alicante (region of south-east Spain). A maximum of six matches per team were played in two days. A total of 46 observations were included in the study. Some matches reached the third set (shoot out), but this additional set was not considered in the analysis.

A global positioning system (GPS, Spi Pro X, GPSports, Canberra, Australia) was used on each player’s back to analyze their physical demands during the match, in stable atmospheric and signal reception conditions. Positional variables (distance and speed) were registered at a sampling frequency of 15 Hz. On the other hand, acceleration was recorded at 100 Hz through a triaxial accelerometer. A heart rate (HR) band was also used by each player (Polar Electro, Kempele, Finland). A standardized warm-up was applied before the test with running, mobility, and high-intensity activities [33].

This system consists of three segments: (a) satellite signal reception and location system (spatial); (b) Earth calibration station (control); and (c) GPS receiver (user). The space segment is made up of a network of at least 24 satellites in Medium Earth Orbit (MEO) that emit the signals (30 operational), of which at least four are required for three-dimensional navigation, which includes latitude, longitude and altitude (in the case of the present investigation, information was received from up to 12 satellites) [34]. This navigation system is made possible by the creation of a constellation of GPS satellites. The GPS satellite constellation has satellites in six equally spaced orbital planes (Earth Centered Inertial coordinates—ECI); it also indicates the exact position of the satellites in these orbital planes. The uneven offset of the satellites in each plane is designed to minimize the effect of satellite disruption [35,36]. The control segment is made up of a set of high-precision stations located on the ground. This segment has several objectives: (1) To keep each satellite in its proper orbit through small and infrequent commanded maneuvers; (2) To perform corrections and settings to the satellite clocks and payload as required; (3) To track the GPS satellites and generate and upload the navigation data to each of the GPS satellites; and (4) To manage major relocations in the event of satellite failure to minimize impact [35,36]. However, one problem identified in this segment is the overdetermined system of equations to estimate the location. The skip to the output of the location data from the GPS receivers and the sudden acceleration to very high speeds lead to a large error in the determination of the receiver’s position. Some methods such as the least squares (LS) method that has been presented to solve navigation equations so far generally have low accuracy and high error [37]. On the other hand, an alternative solution was presented by Fursov et al. [38]; these authors proposed the method of forming a formed subsystem using an auxiliary system obtained from the original system by reducing the columns, providing a more precise estimate of the Rational Polynomial Coefficients (RPCs) compared to the Least-Squares Method (LSM) and Least Absolute Deviations (LAD) methods. In this sense, the authors Edgecomb and Norton [39] conclude that both GPS technology and computer-based tracking systems involve systematic errors, overestimating the distance travelled. Nevertheless, such errors are relatively small and predictable, so it is considered that the use of any of these technologies to monitor athletes’ movements should not be prevented [32]. The above-mentioned problem occurs at very high speeds reached by, e.g., aeroplanes [37].

**Figure 1:**
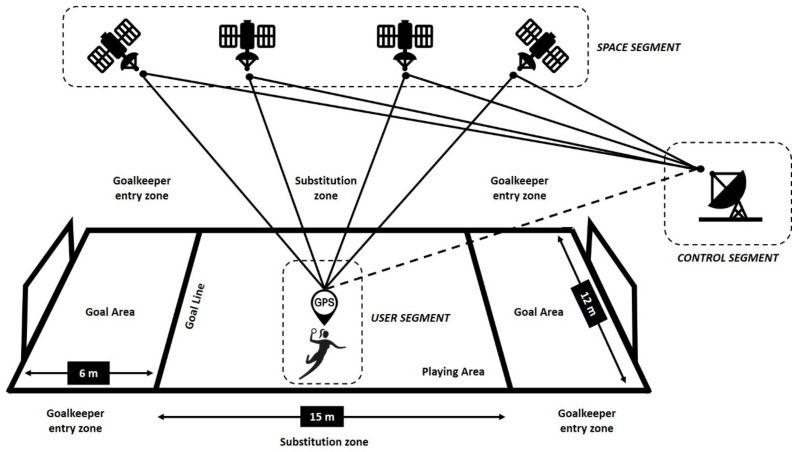
Application of the GPS system in beach handball.

The user segment corresponds to the GPS receivers that receive and decode the signals transmitted by the satellites (Figure 1). All of this satellite navigation is possible thanks to the previous creation of the atomic clock, which is the device that allows the calculation of the time that it takes a radio signal to travel from the satellite to the GPS receiver on Earth [16]. Ultimately, satellites transmit signals to GPS receivers to determine the location, speed, and direction of the devices [17,40].

#### 2.3.1. Movement Patterns

The external load was evaluated according to the distance covered in five speed zones: zone 1: 0–0.4 km·h^−1^; zone 2: 0.5–4 km·h^−1^; zone 3: 4.1–7 km·h^−1^; zone 4: 7.1–13 km·h^−1^; and zone 5: >13.1 km·h^−1^. Speed zone thresholds were considered according to previous studies conducted in beach handball players [8]. In addition, the GPSs attached to the players provided information about the average (V_MEAN_) and maximum (V_MAX_) speed according to the positional parameters (x, y) [33,41]. 

Internal load was monitored by means of Heart Rate (HR) data. The maximum HR (HR_MAX_) of each player was calculated using the formula proposed by Tanaka et al. [42]. Six HR zones were analyzed according to the HR_MAX_ of each player: zone 1: <60% HR_MAX_; zone 2: 60–70% HR_MAX_; zone 3: 70–80% HR_MAX_; zone 4: 80–90% HR_MAX_; zone 5: 90–95% HR_MAX_; and zone 6: >95% HR_MAX_. The average of the HR (HR_MEAN_) was also included in the analysis. Data were evaluated using the software provided by the GPS manufacturer (Team AMS R1 2015, GPSports, Canberra, Australia). 

#### 2.3.2. Muscular Strength

Abalakov jumps and CMJs were performed immediately before and after the match. An infrared system (Optojump Next, Microgate, Bolzano, Italy) was used to obtain the jump height. Beach handball players kept their hands on their hips to avoid the effect of arm movement on jump performance. Every participant performed two different jumps before and after the matches with two minutes of recovery between jumps. The best jump was used for the analysis of the results [33]. 

Upper-body strength was evaluated using a hand dynamometer with adjustable grip (TKK 5001 Grip A; Tokyo, Japan). Beach handball players closed their hands in a 2-s maximum repetition with the dominant hand and the arm totally extended. Two repetitions were performed before and after the matches and the best result (in kg) was included in the statistical analysis [43].

### 2.4. Statistical Analysis

Data are presented as means ± standard deviations. The Kolmogorov–Smirnov distribution test was performed to confirm a normal distribution of the variables. Firstly, physical demand variables were compared between the first set and second set Student’s t-test, using the set (first and second set) as an independent variable and the movement patterns and physislogical parameters as depent variables. Secondly, physical performance test results were compared between the basal situation and the six matches by one-way analysis of variance (one-way ANOVA), using the match as the independent variable and the results of the physical performance tests as the dependent variable. Effect sizes (Cohen’s d, ES) were calculated and defined as follows: trivial, <0.19; small, 0.2–0.49; medium, 0.5–0.79; and large, >0.8. All data were statistically analyzed using SPSS V24.0 for Windows (SPSS Inc., Chicago, IL, USA). The level of significance was set at *p* < 0.05.

## 3. Results

**Figure 2:**
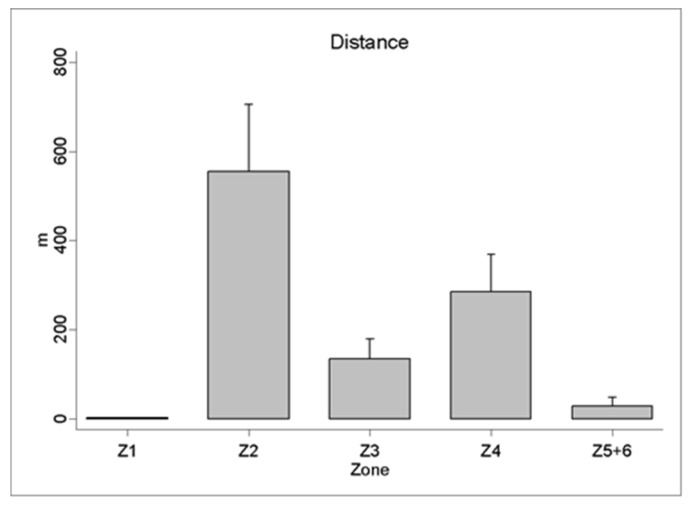
Activity profile during a beach handball game, expressed as the distance covered in different locomotor categories: zone 1: 0–0.4 km·h^−1^; zone 2: 0.5–4 km·h^−1^; zone 3: 4.1–7 km·h^−1^; zone 4: 7.1–13 km·h^−1^; and zone 5: >13.1 km·h^−1^.

**Figure 3:**
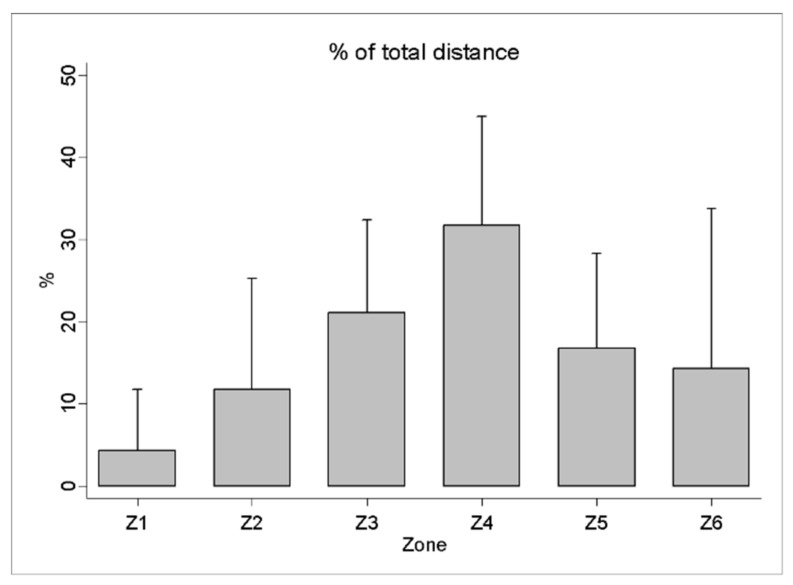
Distribution of heart rate expressed as the percentage of game time taken in the different ranges of HR_MAX_ during a beach handball match.

The analysis of the physical demands of the beach handball players during the matches revealed a total distance covered of 1004.49 ± 258.11 m and a maximum speed of 15.77 ± 1.57 km·h^−1^. The distance travelled in the different speed ranges showed a greater distance in zone 2 (554.50 ± 151.64 m) and a sprint distance (zone 5) of 28.28 ± 19.27 m (Figure 2). In relation to heart rate, the results showed a HR_MEAN_ of 166.44 ± 14.60 b.p.m. and a HR_MAX_ of 193.58 ± 10.68 b.p.m. The accumulated time in the different intensity zones revealed 62.82 ± 14.73% of the time above 80% of the HR_MAX_ (Figure 3).

**Figure 4:**
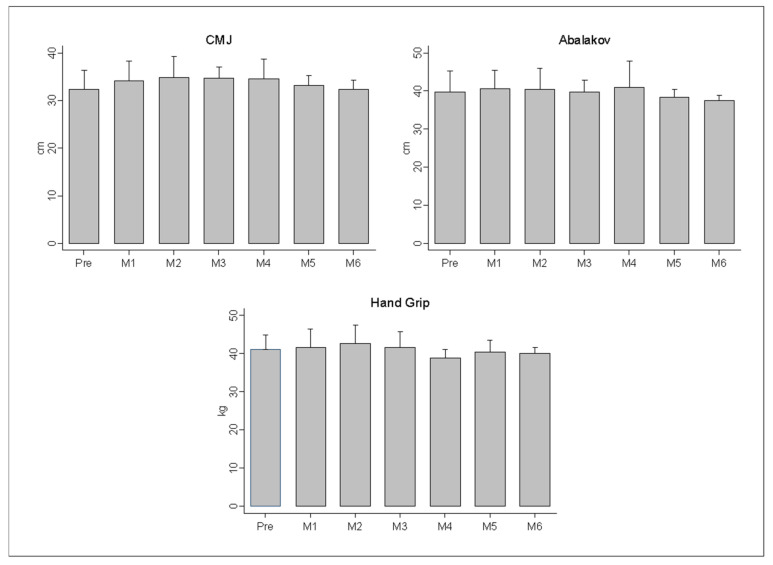
Evolution of the Countermovement Jumps (cm), Abalakov jump (cm), and handgrip (kg) values during the six matches of the tournament in women’s beach handball players.

The analysis of fatigue during a match showed a reduction in total distance (−44.92 m; CI 95%: −87.64 to −2.19; ES: 0.41), the distances covered in zone 3 (−9.95 m; CI 95%: −16.87 to −3.04; ES: 0.50), and zone 4 (−19.15 m; CI 95%: −37.23 to −1.07; ES: 0.50) as well as the V_MEAN_ (−0.21 km·h^−1^; CI 95%: −0.40 to −0.02; ES: 0.40) in the second set (*p* < 0.05; Table 1). The rest of the variables did not show significant differences between the first and the second set (*p* > 0.05). Finally, there were no significant decreases in jumping ability and handgrip during the tournament in beach handball players (*p* > 0.05; Figure 4).

**Table 1:** Activity profile and heart rate values of women beach handball players during a tournament.

	**First Set**			**Second Set**		
Activity Profile						
Total Distance (m)	471.39	±	103.68	426.47	±	112.85 *
Distance Zone 1 (m)	0.66	±	0.57	0.51	±	0.41
Distance Zone 2 (m)	251.37	±	69.81	232.84	±	61.65
Distance Zone 3 (m)	66.79	±	17.95	56.83	±	21.63 *
Distance Zone 4 (m)	140.42	±	33.27	121.27	±	43.82 *
Distance Zone 5 (m)	12.05	±	11.10	14.89	±	14.28
V_MEAN_ (km·h^−1^)	2.54	±	0.56	2.33	±	0.50 *
V_MAX_ (km·h^−1^)	14.63	±	1.71	14.91	±	2.30
Heart Rate						
HR_MEAN_ (b.p.m.)	168.92	±	14.48	171.22	±	16.33
HR_MAX_ (b.p.m.)	190.30	±	10.24	191.84	±	12.77
HR Zone 1 (%)	4.08	±	9.19	2.52	±	6.67
HR Zone 2 (%)	8.74	±	12.48	9.13	±	16.01
HR Zone 3 (%)	18.24	±	14.28	17.03	±	15.60
HR Zone 4 (%)	35.16	±	18.84	31.04	±	17.02
HR Zone 5 (%)	17.41	±	14.04	21.54	±	15.60
HR Zone 6 (%)	16.37	±	23.57	18.74	±	23.07

* Significant differences between first and second set (*p* < 0.05).

## 4. Discussion

This study is the first investigation that analyses the physical demands of beach handball players during an official competition (Arena Handball Tour^®^). For this analysis, GPS devices have been used [44] with the intention of comparing the physical demands between the first and second sets of the matches. The analysis of variance revealed a reduction in the distance covered at high intensity during the second set. However, the internal load shows no difference between the first and second sets. Likewise, the cumulative load of a congested period of matches did not show a significant effect on the jumping ability and upper-body strength of beach handball players. Previous studies that have investigated the kinematic and physiological demands in beach handball during match simulations have described the determining variables in simulated or unofficial matches [8,9,45]. In contrast, the data obtained in the present investigation were collected in official matches of a national competition in Spain. Therefore, the interpretation of the results must be analyzed with caution due to the differences between the internal and external load required in an official competition and that of training matches or unofficial matches.

The total distance covered by the players was 1004.49 ± 258.11 m, 1.34% lower than that reported by Pueo et al. [8] with 1018 ± 222 m. Depending on the set, the results showed 471.39 ± 103.68 m covered during the first set and 426.47 ± 112.85 m during the second set. These results were superior to the data obtained by Zapardiel and Asín-Izquierdo [9] with a mean of 369.7 ± 158.4 (m), and by Gutiérrez-Vargas et al. [45] with 332.2 ± 134.7 (m) travelled in the first set and 281.2 ± 87.7 (m) in the second set, and lower than those achieved by Pueo et al. [8] with 614 ± 145 (m) in the first set and 504 ± 130 (m) in the second. The differences in the distances covered may be due to the heterogeneity in the study samples or the differences in the playing surfaces that present a variable and non-standardized depth, the mechanical properties of sand being one of the determining variables of the performance in the players [46]. The analysis of the distance travelled in the different speed zones does not follow the same methodological definition due to the differences in the definition of the speed ranges used in the studies. These differences are based on the creation of the speed ranges through the maximum speed of each player, as Pueo et al. [8] establish 18.1 km·h^−1^ as the maximum speed following the studies of Cummins et al. [18]. Zapardiel and Asín-Izquierdo [9] establish the limit at 15 km·h^−1^ based on the previous results obtained in their own study. The present investigation follows a similar methodology to Zapardiel and Asín-Izquierdo [9] when obtaining a maximum speed during the competition of 15.77 ± 1.57 km·h^−1^. The study of the distance in the speed zones indicates how, in zones 2 and, 4 the greater distance travelled by the players is obtained, describing the kinematic demands of an intermittent discipline with two differentiated work speed zones. The study of the distance travelled in the game sets reports significant differences between the first and the second set in zones 3 and 4. In the same line of research, although with different speed zones, Pueo et al. [8] show significant differences in the distances covered at low intensity (zone 1) between the first and second sets. However, the two most representative intensity zones (2 and 4), which account for 83.1% of the total distance covered, show the intermittence of this discipline, favored by the unlimited substitutions of the players during each set and the short duration of the sets.

The influence of the playing period on the performance of the players has been studied in other sports, such as futsal [47,48], basketball [49], rugby 7 [50,51], hockey [52,53], and handball [54], with the aim of studying the effect of fatigue on the physical demands of players during competition. A differentiation of beach handball compared to other collaboration-opposition sports that use common space and simultaneous practice would be the regulatory conditioner with respect to the final score that is determined by the independence of the result of each set. This aspect will be a key factor in determining the fatigue of the players. In the present study, the analysis of fatigue during the match showed a reduction in the total distance between the first and second sets of a 9.5% difference, in the distances covered in zone 3 with a 14.9% difference in the 1st and 2nd sets, and in zone 4 with a 13.6% difference in the 1st and 2nd sets. In addition, in the V_MEAN_, there was an 8.2% difference between sets. Pueo et al. [8] continue this line of work and their results coincide with the present article, since, in the second set, the distance travelled at low intensity is significantly lower than that travelled in the first set, while the distance travelled at high intensity does not reveal differences between both sets. At the internal load level, the variables HR_MAX_ 193.58 ± 10.68 and HR_MEAN_ 166.44 ± 14.6 of the present study coincide with the results presented by Zapardiel and Asín-Izquierdo [9], where it is observed that beach handball is a high-intensity discipline where the longest activity time is accumulated above 80% of the HR_MAX_ (62.82 ± 14.73%). Previous studies show a similar distribution in relation to the accumulated time in the different intensity zones, highlighting the intermittent nature of this sport [55].

Edwards et al. [56], define the valid tools to monitor fatigue and propose the evaluation of vertical jumps as an easy tool to administer with minimal fatigue in its execution, following the line of research in other disciplines, such as athletics [26], football [57], or basketball [58], where the jump test (CMJ) has been used as a predictor of performance or fatigue. Furthermore, the importance of this action in the performance of beach handball players shows the need to evaluate the effect of fatigue on the jumping ability of athletes. The results of this study show an absence of fatigue of the players after the matches, revealing similar results in the jumping ability of the players during a congested period of matches, unlike other sports such as rugby [59], where up to 6% loss of jumping post-match was found, or in basketball with a decrease in peak power output calculated through a CMJ of 0.5% [60]. In handball, likewise, there is a decrease in this post-match variable of 5.2% (*p* > 0.01) [61]. In other sports such as beach football, an improvement in jumping ability is reported after the game [62]. The explanation of these results may be due to the unstable surface, such as sand, as Impellizzeri et al. [63] explained in their study on plyometric training on this surface. In this study, it is demonstrated that the physiological response leads to an improvement in neuromuscular activation factors and less muscle pain (less muscle soreness) during exercise on beach sand. Lastly, the analysis of fatigue in beach handball players revealed similar results in jumping ability and upper-body strength during a congested period of matches within two days. The lack of research carried out in the context of official championships makes the comparison impossible. A possible explanation for this lack of fatigue would be the competition system used in this sport, which is based on different matches played during two consecutive days. This would imply that sufficient accumulated fatigue is not reached due to the competition formats used in this sport [11], since unlike other collaboration-opposition sports, beach handball is characterized by the dispute of two independent sets of short duration with intermittent pause periods, a 5 min rest, unlimited substitutions, and clearly differentiated attack-defense phases that favor the recovery of the players.

## 5. Conclusions

The main finding of the present manuscript is that 53% of the distance travelled is done at speeds between 0.5 and 4 km/h, and 30% of the distance is between 7.1 and 13 km/h, adding up to 83% of the total distance covered, which shows the intermittence of efforts that beach handball involves at high intensity, as reflected in the analysis of the internal load with 62.82 ± 14.73% of the time above 80% of the HR_MAX_. Regarding the difference between sets, the distance travelled in zone 4 stands out, with this difference being significant in defining the kinematic demand of the discipline. The results of this study demonstrate that a congested period of matches does not alter the jumping ability or upper-body strength of beach handball players. The dynamics of the game itself, as well as the unlimited possibility of substitutions, are the key elements to maintaining performance during the competition. This aspect will allow for better training planning with greater specificity and improvement of beach handball players.

## Data Availability

The raw data supporting the conclusions of this article will be made available by the authors, without undue reservation, to any qualified researcher.
